# Vancomycin concentrations in the cervical spine after intravenous administration: results from an experimental pig study

**DOI:** 10.1080/17453674.2018.1501548

**Published:** 2018-08-06

**Authors:** Mats Bue, Pelle Hanberg, Mikkel Tøttrup, Maja B Thomassen, Hanne Birke-Sørensen, Theis M Thillemann, Torben L Andersson, Kjeld Søballe

**Affiliations:** 1 Department of Orthopaedic Surgery, Horsens Regional Hospital, Horsens;; 2 Orthopaedic Research Unit, Aarhus University Hospital, Aarhus;; 3 Department of Orthopaedic Surgery, Randers Regional Hospital, Randers;; 4 Department of Orthopaedic Surgery, Aarhus University Hospital, Aarhus;; 5 Department of Clinical Biochemistry, Aarhus University Hospital, Aarhus, Denmark

## Abstract

Background and purpose — Vancomycin may be an important drug for intravenous perioperative antimicrobial prophylaxis in spine surgery. We assessed single-dose vancomycin intervertebral disc, vertebral cancellous bone, and subcutaneous adipose tissue concentrations using microdialysis in a pig model.

Material and methods — 8 female pigs received 1,000 mg of vancomycin intravenously as a single dose over 100 minutes. Microdialysis probes were placed in the C3–C4 intervertebral disc, C3 vertebral cancellous bone, and subcutaneous adipose tissue, and vancomycin concentrations were obtained over 8 hours. Venous blood samples were obtained as reference.

Results — Ranging from 0.24 to 0.60, vancomycin tissue penetration, expressed as the ratio of tissue to plasma area under the concentration-time curve from 0 to the last measured value, was incomplete for all compartments. The lowest penetration was found in the intervertebral disc. The time to a mean clinically relevant minimal inhibitory concentration (MIC) of 4 µg/mL was 3, 17, 25, and 156 min for plasma, subcutaneous adipose tissue, vertebral cancellous bone, and the intervertebral disc, respectively. In contrast to the other compartments, a mean MIC of 8 µg/mL was not reached in the intervertebral disc. An approximately 3-times longer elimination rate was observed in the intervertebral disc in comparison with all the other compartments (p < 0.001), and the time to peak drug concentration was higher for all tissues compared with plasma

Interpretation — Preoperative administration of 1,000 mg of vancomycin may provide adequate vancomycin tissue concentrations with a considerable delay, though tissue penetration was incomplete. However, in order also to achieve adequate intervertebral disc concentrations in all individuals and accommodating a potentially higher MIC target, supplemental application of vancomycin may be necessary.

Postoperative surgical site infections following spine surgery can have devastating complications such as mechanical fixation failure, neurological injury, pseudoarthrosis, and sepsis (Fang et al. 2005). The reported incidence of postoperative spondylodiscitis ranges between 1% and 4% and may be even higher when instrumentation is applied (Gaynes et al. 2001, Deyo et al. 2004, Fang et al. 2005, Smith et al. 2011). Perioperative antimicrobial prophylaxis plays a key role in lowering the risk of postoperative spondylodiscitis. The antimicrobial effect relies not only on its sensitivity against the invading bacteria, but also on adequate target site concentrations. For vancomycin, tissue targets for prevention of postoperative spondylodiscitis have not been established. Nonetheless, it seems prudent to maintain tissue concentrations that, as a minimum, exceed minimal inhibitory concentrations (MIC) of relevant bacteria throughout surgery and until a few hours after the incision is closed (Mangram et al. 1999, Whiteside 2016). Vancomycin may become increasingly important as perioperative antimicrobial prophylaxis in spine surgery due to the increasing incidence of methicillin-resistant Staphylococcus aureus (MRSA) infections (Al-Nammari et al. 2007, Gouliouris et al. 2010). However, routine use of vancomycin is not indicated due to the risk of resistance development (Mangram et al. 1999, Bryson et al. 2016). Incomplete vancomycin penetration has recently been reported for a number of tissues (Bue et al. 2018a, b). Given the avascular nature of the intervertebral disc, vancomycin penetration may be inadequate for optimal prevention of postoperative spondylodiscitis.

It is challenging to determine antimicrobial spine tissue concentrations. Intervertebral disc and bone concentrations of vancomycin have almost only been evaluated using bone and disc tissue samples and discectomy (Scuderi et al. 1993, Conaughty et al. 2006, Landersdorfer et al. 2009, Komatsu et al. 2010). However, these methods suffer from methodological challenges, which makes it difficult to relate the results to relevant pharmacokinetic/pharmacodynamic endpoints (Landersdorfer et al. 2009, Pea 2009). Recently, microdialysis has evolved as a promising method for sampling various antimicrobials in different types of tissues, including bone and the intervertebral disc (Stolle et al. 2004, Bue et al. 2015, Tottrup et al. 2015, Hanberg et al. 2016, Bue et al. 2018a, b).

We assessed single-dose vancomycin concentrations in the C3–C4 intervertebral disc, the C3 vertebral cancellous bone, and subcutaneous adipose tissue using microdialysis in a pig model mimicking a perioperative situation. Tissue penetration ratios, expressed as the ratio of tissue to plasma area under the concentration-time curve from 0 to the last measured value (AUC_tissue_/AUC_plasma_), and time to mean MICs of 2, 4, and 8 µg/mL were the primary endpoints. The secondary endpoints were pharmacokinetic parameters: the area under the concentration-time curves (AUC_0–last_), peak drug concentration (C_max_), time to C_max_ (T_max_), and half-life (T_1/2_).

## Material and methods

This study was conducted at the Institute for Clinical Medicine, Aarhus University Hospital, Aarhus, Denmark. All chemical analyses were performed at the Department of Clinical Biochemistry, Aarhus University Hospital, Aarhus, Denmark.

### Overview

8 female pigs were included in the study (Danish Landrace Breed; weight 78–82 kg). Vancomycin was administered intravenously as a single dose of 1,000 mg over 100 min, and sampling was conducted over 8 hours starting at the beginning of vancomycin infusion. Vancomycin concentrations were obtained using microdialysis in the C3–C4 intervertebral disc, the C3 vertebral cancellous bone, and subcutaneous adipose tissue.

### Anesthesia and surgical procedures

The pigs were kept under general anesthesia using a combination of fentanyl (0.35–0.5 mg/h, continuous infusion) and propofol (500–600 mg/h, continuous infusion) during the surgery and the sampling period. Arterial pH was monitored throughout the study and kept in the range of 7.36–7.47 by regulating ventilation. Blankets were used to keep the core temperatures within the range of 36.2–39.1 °C.

Immediately after induction of anesthesia, the surgical procedures were initiated. With the pig in supine position, and under fluoroscopic guidance, the C2–C4 vertebrae were exposed via an anterolateral incision. At approximately 45° to the sagittal plane, a drill hole with a diameter of 2 mm and a depth of 25 mm was created in the middle of C3. Parallel to this drill hole, a Kirschner wire with a fixating device (PEBAX, M Dialysis AB, Stockholm, Sweden) was drilled into the caudal part of C2. A microdialysis probe (membrane length 20 mm) was inserted into a splittable introducer with the membrane protruding approximately 30 mm from the tip of the introducer. This probe-introducer setup was then placed in the drill hole in C3 and fixed to the fixating device; an endo clip was then attached to the introducer. These steps were taken to avoid subsequent displacement of the probe. At the same angle, a splittable introducer with a needle was introduced into the intervertebral disc between C3 and C4 parallel to, and in the middle of, the adjacent endplates. After the annulus fibrosus was penetrated, the needle was retracted, and the introducer was carefully advanced into the nucleus pulposus until resistance from the opposite wall of the annulus fibrosus was felt. A microdialysis probe (membrane length 10 mm) was then placed in the introducer, and the splittable introducer was retracted until the entire membrane of the probe was exposed in the intervertebral disc. The probe was attached to the introducer with endo clips. Fluoroscopy was used to assess correct location of the probes in the C3 vertebral body and the C3–C4 intervertebral disc. In addition to the bone and intervertebral disc probes, a subcutaneous adipose tissue probe (membrane length 20 mm) was placed in the lateral part of the right thigh, based on the manufacturer’s guidelines.

### Microdialysis and sampling procedures

A detailed description of microdialysis can be found elsewhere (Muller 2002, Joukhadar and Muller 2005). Briefly, microdialysis is a probe-based method that allows for serial sampling of water-soluble molecules from the extracellular fluid in the tissue of interest by means of a semipermeable membrane at the tip of the microdialysis probe (Joukhadar et al. 2001, Hutschala et al. 2013). Due to continuous perfusion of the probe, a non-equilibrium diffusion of molecules following the concentration gradient will occur. Consequently, the concentration in the dialysate represents only a fraction of the true tissue concentration, which is expressed as the relative recovery. Thus, relative recovery must be determined to calculate the absolute tissue concentrations. In the present study, all the microdialysis probes were individually calibrated at the end of the study using the retrodialysis by drug method (Scheller and Kolb 1991). The relative recovery was calculated using the following Equation 1:

(1) $${}^{\text{Relative recovery }\left(\%\right)\;=\;\text{1}00\;\times \;\left(\text{1}–\text{Cdialysate}/\text{Cperfusate}\right)}$$

where C_dialysate_ is the concentration (µg/mL) in the dialysate and C_perfusate_ is the concentration (µg/mL) in the perfusate.

Absolute, extracellular concentrations (µg/mL), C_tissue_, were calculated by correcting for relative recovery using the following Equation 2:

(2) $${}^{\text{Ctissue}\;=\;\text{1}00\;\times \;\text{Cdialysate}/\text{Relative recovery }\left(\%\right)}$$

The microdialysis system consisted of CMA 107 precision pumps (M Dialysis AB, Stockholm, Sweden) and CMA 70 probes (membrane length 20 mm and 10 mm, molecular cut-off 20 kilo Daltons). All the microdialysis probes were perfused with 0.9% NaCl at a perfusion rate of 1 µL/min throughout the sampling time. Following a 30-min tissue equilibration period after placement of the microdialysis probes, 1,000 mg of vancomycin was administered intravenously over 100 min starting at time zero. For the first 2 hours, dialysates were harvested at 40-min intervals and, thereafter, at 60-min intervals for the following 6 hours, resulting in a total of nine samples over 8 hours. The relative recovery-corrected dialysate concentrations were ascribed to the midpoint of each sampling interval. Venous blood samples were harvested from a central venous catheter at the midpoint of the same 9 sampling intervals. When the last dialysate was collected, all the probes were individually calibrated using 0.9% NaCl with vancomycin at a concentration of 300 µg/mL by collecting 60-min samples. This high calibration concentration of vancomycin was chosen to minimize the influence of the residual local tissue concentrations. After the last dialysates were collected, the pigs were killed using pentobarbital.

The dialysates were immediately placed in a –80 °C freezer until analysis. The venous blood samples were stored at 5 °C for a maximum of eight hours before being centrifuged at 3,000 g for 10 minutes. The plasma aliquots were then frozen and stored at –80 °C until analysis.

### Chemical analysis of vancomycin

The vancomycin concentrations in the dialysates were quantified using ultra-high-performance liquid chromatography as previously described (Bue et al. 2015). The quantification limit was defined as the lowest concentration with intra-run CV <20%; it was found to be 0.05 µg/mL. The free concentration of vancomycin in plasma was measured with a homogeneous enzyme immunoassay technique using the Siemens Chemistry XPT platform (Advia Chemistry, Erlangen, Germany). Intra-run (total) imprecisions for the assay were ±1.2 µg/mL (2SD) at 6.6 µg/mL and ±3.7 µg/mL (2SD) at 29.1 µg/mL.

### Pharmacokinetic analysis and statistics

Using Microsoft Excel (Microsoft Corp, Redmond, WA, USA), the time to mean clinically relevant MICs of 2, 4, and 8 µg/mL was estimated using linear interpolation. The pharmacokinetic parameters, AUC_0–last_, C_max_, T_max_, and T_1/2_, were determined separately for each compartment for each pig by non-compartmental analysis using the pharmacokinetic-series of commands in Stata (v. 14.1, StataCorp LLC, College Station, TX, USA). The AUC_0–last_ was calculated using the trapezoidal rule. The tissue AUC_0–last_ to plasma AUC_0–last_ ratio (AUC_tissue_/AUC_plasma_) was calculated as a measure of tissue penetration. C_max_ was calculated as the maximum of all the recorded concentrations and T_max_ was calculated as the time to C_max_. T_1/2_ was calculated as ln(2)/λ_eq_, where λ_eq_ is the terminal elimination rate constant estimated by linear regression of the log concentration on time. These pharmacokinetic parameters were obtained in all 4 compartments from the same pig and a mixed model for repeated measurements had compartments as fixed effect and subject identification variable as a random effect was applied. Also, distinct residual variance was assumed within each compartment. The normality of the residuals was estimated using a quantile–quantile (QQ) plot for the residuals and the homogeneity of the residual variance was checked by plotting residuals vs. best linear unbiased prediction estimates. The normality of the estimated random effects was checked using a QQ plot of the estimated random effects. A correction for degrees of freedom due to small sample size was handled using the Kenward–Roger approximation method. The F-test was used to determine the overall comparisons between the compartments and the t-test was used to determine pairwise comparisons. A p-value <0.05 was considered to be significant. Statistical analyses were also performed using Stata. Values below the lower limit of quantification were set to zero. The means and 95% CI of AUC_0–last_, C_max_, T_max_, and T_1/2_ are presented in the Table.

**Table:** Table: Pharmacokinetic parameters for plasma, subcutaneous adipose tissue, vertebral cancellous bone, and intervertebral disc

Tissue	AUC_0–last_ (min µg/mL)	C_max_ (µg/mL)	T_max_ (min)	T_1/2_ (min)	AUC_tissue/_AUC_plasma_
Plasma (unbound)	7,880 (7164–8597) ^b^	40.0 (35.7–44.3) ^b^	75 (61–89)	325 (99–552)	
Subcutaneous adipose tissue	4,719 (4002–5436)	18.0 (14.1–21.9)	113 (96–129)	224 (197–250)	0.60 (0.48–0.72)
Vertebral cancellous bone	3,677 (2960–4393) ^c^	12.3 (9.6–15.0) ^c^	159 (134–184)	271 (227–315)	0.46 (0.40–0.53)
Intervertebral disc	1,983 (1237–2729) ^d^	6.6 (3.6–9.6)4	270 (187–353)	933 (527–1,339) ^e^	0.24 (0.17–0.31)
p-value**^a^**	< 0.001	< 0.001	–	< 0.008	

Values are given as means (95% confidence interval).

AUC_0–last_, area under the concentration-time curve from 0 to the last measured value; C_max_, peak drug concentration; T_max_, time to C_max_; T1/2, half-life at β-phase; AUC_tissue_/AUC_plasma_, tissue penetration expressed as the ratio of AUC_tissue_/AUC_plasma_.

**^a^**Overall comparison using F test for plasma (unbound), subcutaneous adipose tissue, vertebral cancellous bone, and intervertebral disc.

**^b^**p < 0.001 for all comparisons between plasma and the other compartments.

**^c^**p < 0.01 for comparison with subcutaneous adipose tissue.

**^d^**p < 0.01 for comparison between intervertebral disc and the other compartments.

**^e^**p < 0.001 for all comparisons between intervertebral disc and the other compartments.

### Ethics, funding, and potential conflicts of interest

The study was approved by the Danish Animal Experiments Inspectorate and carried out according to existing laws (license No. 2017/15-0201-01184).

This work was supported by unrestricted grants from the Augustinus Foundation, the Lippmann Foundation, the Knud and Edith Eriksens Memorial Foundation, the Søster and Verner Lipperts Foundation, and the Health Research Fund of Central Denmark Region. No competing interests were declared.

## Results

**Figure 1.**
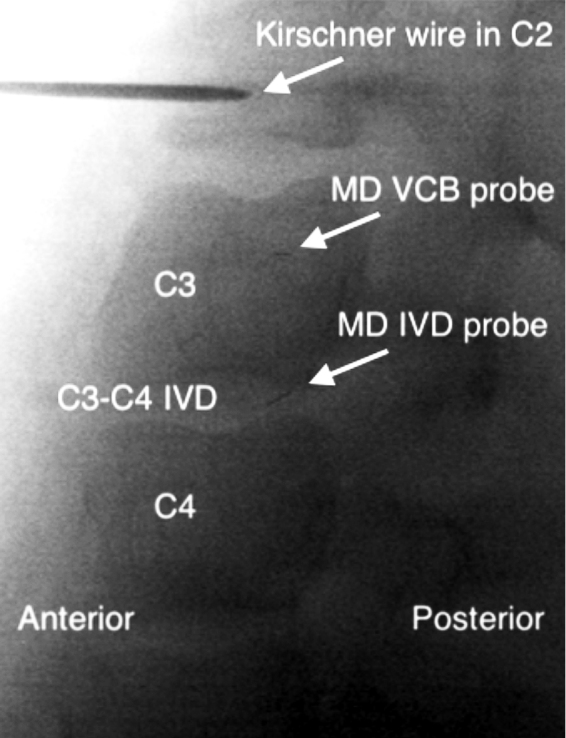
Representative fluoroscopic image showing the location of the microdialysis (MD) probes in a sagittal view: the Kirschner wire with the fixating device in the C2 vertebral body, C3 and C4 vertebral body, C3–C4 intervertebral disc (IVD), the gold thread within the microdialysis probe membrane tip in the vertebral cancellous bone (VCB) and intervertebral disc.

All 8 pigs completed the study. Except for one malfunctioning intervertebral disc probe, data were obtained from all probes. Fluoroscopy confirmed correct placement of all of the probes (Figure 1). Mean (SD) relative recovery for the intervertebral disc, vertebral cancellous bone, and subcutaneous adipose tissue was 16% (6), 36% (4), and 33% (5), respectively.

Tissue penetration (AUC_tissue_/AUC_plasma_) of vancomycin (95% CI) was incomplete for the subcutaneous adipose tissue 0.60 (0.48–0.72), vertebral cancellous bone 0.46 (0.40–0.53), and intervertebral disc 0.24 (0.17–0.31). After 15 min, a mean concentration of 2 µg/mL (MIC) was reached in all compartments. The time to a mean MIC of 4 µg/mL was 3, 17, 25, and 156 min for plasma, subcutaneous adipose tissue, vertebral cancellous bone, and the intervertebral disc, respectively. A mean MIC of 8 µg/mL could not be reached in the intervertebral disc, whereas it was reached after 7, 37, and 81 min in plasma, subcutaneous adipose tissue, and vertebral cancellous bone, respectively.

**Figure 2.**
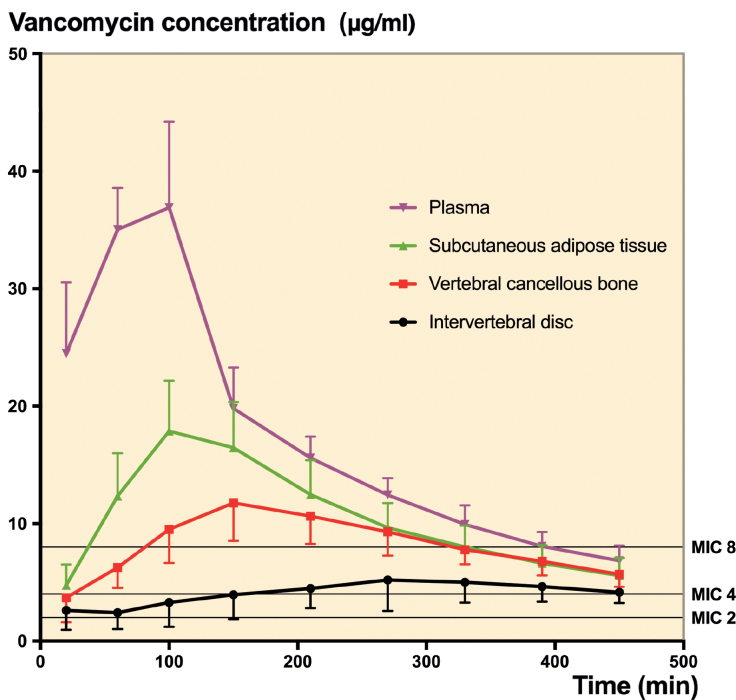
Mean concentration-time profiles for plasma, subcutaneous adipose tissue, vertebral cancellous bone, and the intervertebral disc. Bars represent 95% confidence intervals. MICs of 2, 4, and 8 µg/mL are also inserted.

The vancomycin tissue and plasma concentration-time profiles are shown in Figure 2. The pharmacokinetic parameters are presented in the Table. C_max_ (95% CI) was 6.6 µg/mL (3.6–9.6) for the intervertebral disc, 12 µg/mL (9.6–15) for vertebral cancellous bone, 18 µg/mL (14–22) for subcutaneous adipose tissue, and 40 µg/mL (36–44) for plasma. The T_max_ findings revealed delayed tissue penetration, particularly to the intervertebral disc and vertebral cancellous bone. Furthermore, T_1/2_ was approximately 3 times longer in the intervertebral disc in comparison with the other compartments (p < 0.001). Finally, AUC_0–last_ and C_max_ were lower in the intervertebral disc than in the vertebral cancellous bone (p < 0.01).

## Discussion

To our knowledge, this is the first study to investigate single-dose vancomycin intervertebral disc and vertebral cancellous bone concentrations using microdialysis. Insufficient perioperative antimicrobial target site penetration might play an important role for the rather high incidence of postoperative spondylodiscitis. A key finding of this study was therefore incomplete and delayed intervertebral disc and vertebral cancellous bone penetration of vancomycin, with the lowest and most delayed penetration found in the intervertebral disc. However, using standard recommendations for prevention of surgical site infections and planktonic MICs of commonly encountered bacteria in spine surgery (0.5–4 µg/mL), adequate mean concentrations were achieved in all compartments, although a considerable delay was found in the intervertebral disc (Gouliouris et al. 2010, EUCAST 2017). Advantageously for a perioperative prophylactic setting, it should be noted that an approximately 3 times longer vancomycin elimination rate was found in the intervertebral disc in comparison with the other compartments. Thus, if vancomycin is administrated in due time, adequate intervertebral disc concentrations may be sustained throughout even long surgical procedures and for some time after. This makes vancomycin attractive because it continues to kill bacteria even after surgery has ended. On the other hand, our data suggest a rather narrow or no margin at all in individuals with low intervertebral disc concentrations to bacteria exhibiting high MICs. Moreover, in the case of MRSA, increasing vancomycin MICs have been demonstrated over the last decades (Steinkraus et al. 2007). The traditional target recommendations for prevention of surgical site infections lack scientific evaluation and may in fact be insufficient for spine surgery, particularly when considering the possible devastating complications of infection in spine surgery. Higher and prolonged tissue concentrations cannot be achieved by means of increasing intravenous vancomycin doses as this is restricted by toxicity (Rybak et al. 2009). Consequently, our findings call for some considerations regarding vancomycin dosing, timing, and administration in the perioperative spine setting. Preoperative administration of 1,000 mg of vancomycin may provide adequate vancomycin tissue concentrations with a considerable delay. However, in order also to achieve adequate intervertebral disc concentrations in all individuals and accommodating a potentially higher MIC target, supplemental application (e.g. as perioperative powder) of vancomycin may be necessary (Murphy et al. 2017).

Incomplete and heterogeneous tissue distribution of antimicrobials has been demonstrated for a diverse combination of drugs and tissues and under different conditions, including infections (Joukhadar et al. 2001, Joukhadar and Muller 2005, Schintler et al. 2009, Hutschala et al. 2013, Tottrup et al. 2015, 2016). In our study, the tissue penetration ratios for vancomycin were lower for all tissues in comparison with an analogous cefuroxime study (Hanberg et al. 2016). These findings suggest that the choice of antimicrobial prophylaxis should not only be based on the characteristics of the infectious bacteria and plasma pharmacokinetics, but also on the tissue pharmacokinetics for the specific drug and the specific setting. In terms of infected tissue, it has previously been shown that vancomycin bone penetration may decrease with progression of an infection (Bue et al. 2018a). This emphasizes the need for spine tissue pharmacokinetic studies of relevant antimicrobials under different conditions.

The penetration of vancomycin into a human intervertebral disc may vary from our findings in the study, as juvenile pigs (aged 5 months) differ from adult humans in several ways (Alini et al. 2008). First, blood vessels in the human annulus fibrosus are only present in the first part of life; thereafter, the perfusion relies upon only diffusion from the endplates (Roberts et al. 2006, Gouliouris et al. 2010). Second, the intervertebral disc is thinner in pigs than in humans, indicating shorter diffusion distances (Alini et al. 2008). Third, the body mass and weight-bearing impact on the vertebral bodies and intervertebral disc differs between humans and pigs. Moreover, higher vancomycin concentrations have been demonstrated in pig bone and tissue concentrations in comparison with male patients undergoing total knee replacement surgery (Bue et al. 2015, Bue et al. 2018b).

Until now, vancomycin bone and intervertebral disc concentrations have only been assessed using bone and disc tissue samples and discectomy (Scuderi et al. 1993, Conaughty et al. 2006, Landersdorfer et al. 2009, Komatsu et al. 2010). In contrast, microdialysis allows for serial sampling of the unbound extracellular concentrations of drug in bone and in the intervertebral disc, and it provides dynamic concentration-time profiles (Joukhadar and Muller 2005, Hanberg et al. 2016). Therefore, the pharmacokinetic parameters obtained by microdialysis are useful for evaluating pharmacodynamic/pharmacokinetic targets. In addition to the inherent inter-species limitations of a porcine study, a certain methodological aspect of the microdialysis approach should be considered when evaluating microdialysis data. Thus, to obtain absolute tissue concentrations, the actual measured concentrations are corrected for relative recovery. This leads to a magnification of the variations associated with pre-analytical sample handling and chemical assay. These variations will increase exponentially as the relative recovery decreases. The variances in plasma and tissue pharmacokinetics found in the present study were comparable, indicating acceptable precision of the measurements within the biological variation.

In summary, vancomycin penetration into healthy pig intervertebral disc and vertebral cancellous bone was found to be incomplete and delayed, with the lowest and most delayed penetration to the intervertebral disc. However, applying standard recommendations for prevention of postoperative spondylodiscitis, preoperative administration of 1,000 mg of vancomycin may provide adequate vancomycin tissue concentrations with a considerable delay. Nonetheless, in order also to achieve adequate intervertebral disc concentrations in all individuals and accommodating a potentially higher MIC target, supplemental application of vancomycin may be necessary. Validation of these findings in a clinical setting is warranted.

MB, PH, MT, MBT, HBS, TMT, and KS initiated and designed the study. MB, MBT, and PH conducted the surgery and MB placed all the probes. MB, MBT, and PH collected the data. TLA performed the chemical analyses. Statistical analysis and interpretation of data was done by MB, PH, MT, HBS, TMT, TLA, and KS. All authors drafted and revised the manuscript.

*Acta* thanks Volker Alt and Ivar Rossvoll for help with peer review of this study.

## References

[CIT0001] Al-Nammari S S, Lucas J D, Lam KS Hematogenous methicillin-resistant Staphylococcus aureus spondylodiscitis. Spine (Phila Pa 1976) 2007; 32(22): 2480–6. 18090089 10.1097/BRS.0b013e318157393e

[CIT0002] Alini M, Eisenstein S M, Ito K, Little C, Kettler A A, Masuda K, et al. Are animal models useful for studying human disc disorders/degeneration? Eur Spine J 2008; 17(1): 2–19. 17632738 10.1007/s00586-007-0414-y PMC2365516

[CIT0003] Bryson D J, Morris D L, Shivji F S, Rollins K R, Snape S, Ollivere B J Antibiotic prophylaxis in orthopaedic surgery: difficult decisions in an era of evolving antibiotic resistance. Bone Joint J 2016; 98-B(8): 1014–19. 27482011 10.1302/0301-620X.98B8.37359

[CIT0004] Bue M, Birke-Sorensen H, Thillemann T M, Hardlei T F, Soballe K, Tottrup M Single-dose pharmacokinetics of vancomycin in porcine cancellous and cortical bone determined by microdialysis. Int J Antimicrob Agents 2015; 46(4): 434–8. 26260192 10.1016/j.ijantimicag.2015.06.014

[CIT0005] Bue M, Hanberg P, Koch J, Jensen L K, Lundorff M, Aalbaek B, et al. Single-dose bone pharmacokinetics of vancomycin in a porcine implant-associated osteomyelitis model. J Orthop Res 2018a; 36(4): 1093–1098 29058823 10.1002/jor.23776

[CIT0006] Bue M, Tottrup M, Hanberg P, Langhoff O, Birke-Sorensen H, Thillemann T M, et al. Bone and subcutaneous adipose tissue pharmacokinetics of vancomycin in total knee replacement patients. Acta Orthop 2018b; 89(1): 95–100 28914105 10.1080/17453674.2017.1373497 PMC5810840

[CIT0007] Conaughty J M, Chen J, Martinez O V, Chiappetta G, Brookfield K F, Eismont F J Efficacy of linezolid versus vancomycin in the treatment of methicillin-resistant Staphylococcus aureus discitis: a controlled animal model. Spine (Phila Pa 1976) 2006; 31(22): E830–2. 17047530 10.1097/01.brs.0000241065.19723.13

[CIT0008] Deyo R A, Nachemson A, Mirza S K Spinal-fusion surgery: the case for restraint. N Engl J Med 2004; 350(7): 722–6. 14960750 10.1056/NEJMsb031771

[CIT0009] EUCAST European Committee on Antimicrobial Susceptibility Testing. European 2017; data from the EUCAST MIC distribution website. https://mic.eucast.org/Eucast2/SearchController/search.jsp?action=init

[CIT0010] Fang A, Hu S S, Endres N, Bradford D S Risk factors for infection after spinal surgery. Spine (Phila Pa 1976) 2005; 30(12): 1460–5. 15959380 10.1097/01.brs.0000166532.58227.4f

[CIT0011] Gaynes R P, Culver D H, Horan T C, Edwards J R, Richards C, Tolson J S Surgical site infection(SSI) rates in the United States, 1992–1998: the National Nosocomial Infections Surveillance System basic SSI risk index. Clin Infect Dis 2001; 33(Suppl. 2): S69–S77. 11486302 10.1086/321860

[CIT0012] Gouliouris T, Aliyu S H, Brown N M Spondylodiscitis: update on diagnosis and management. J Antimicrob Chemother 2010; 65(Suppl 3): iii11–24. 20876624 10.1093/jac/dkq303

[CIT0013] Hanberg P, Bue M, Birke Sorensen H, Soballe K, Tottrup M Pharmacokinetics of single-dose cefuroxime in porcine intervertebral disc and vertebral cancellous bone determined by microdialysis. Spine J 2016; 16(3): 432–8. 26620946 10.1016/j.spinee.2015.11.031

[CIT0014] Hutschala D, Skhirtladze K, Kinstner C, Zeitlinger M, Wisser W, Jaeger W, et al. Effect of cardiopulmonary bypass on regional antibiotic penetration into lung tissue. Antimicrob Agents Chemother 2013; 57(7): 2996–3002. 23587954 10.1128/AAC.02627-12 PMC3697369

[CIT0015] Joukhadar C, Frossard M, Mayer B X, Brunner M, Klein N, Siostrzonek P, et al. Impaired target site penetration of beta-lactams may account for therapeutic failure in patients with septic shock. Crit Care Med 2001; 29(2): 385–91. 11246321 10.1097/00003246-200102000-00030

[CIT0016] Joukhadar C, Muller M Microdialysis: current applications in clinical pharmacokinetic studies and its potential role in the future. Clin Pharmacokinet 2005; 44(9): 895–913. 16122279 10.2165/00003088-200544090-00002

[CIT0017] Komatsu M, Takahata M, Sugawara M, Takekuma Y, Kato T, Ito M, et al. Penetration of linezolid into rabbit intervertebral discs and surrounding tissues. Eur Spine J 2010; 19(12): 2149–55. 20694846 10.1007/s00586-010-1548-x PMC2997209

[CIT0018] Landersdorfer C B, Bulitta J B, Kinzig M, Holzgrabe U, Sorgel F Penetration of antibacterials into bone: pharmacokinetic, pharmacodynamic and bioanalytical considerations. Clin Pharmacokinet 2009; 48(2): 89–124. 19271782 10.2165/00003088-200948020-00002

[CIT0019] Mangram A J, Horan T C, Pearson M L, Silver L C, Jarvis W R Guideline for prevention of surgical site infection, 1999. Centers for Disease Control and Prevention (CDC) Hospital Infection Control Practices Advisory Committee. Am J Infect Control 1999; 27(2): 97–132; quiz 3-4; discussion 96. 10196487

[CIT0020] Muller M Science, medicine, and the future: microdialysis. BMJ 2002; 324(7337): 588–91. 11884326 10.1136/bmj.324.7337.588 PMC1122512

[CIT0021] Murphy E P, Curtin M, Shafqat A, Byrne F, Jadaan M, Rahall E A review of the application of vancomycin powder to posterior spinal fusion wounds with a focus on side effects and infection: a prospective study. Eur J Orthop Surg Traumatol 2017; 27(2): 187–91. 27858249 10.1007/s00590-016-1878-4

[CIT0022] Pea F Penetration of antibacterials into bone: what do we really need to know for optimal prophylaxis and treatment of bone and joint infections? Clin Pharmacokinet 2009; 48(2): 125–7. 19271783 10.2165/0003088-200948020-00003.

[CIT0023] Roberts S, Evans H, Trivedi J, Menage J Histology and pathology of the human intervertebral disc. J Bone Joint Surg Am 2006; 88 Suppl. 2: 10–14. 10.2106/JBJS.F.00019 16595436

[CIT0024] Rybak M, Lomaestro B, Rotschafer J C, Moellering R Jr, Craig W, Billeter M, et al. Therapeutic monitoring of vancomycin in adult patients: a consensus review of the American Society of Health-System Pharmacists, the Infectious Diseases Society of America, and the Society of Infectious Diseases Pharmacists. Am J Health Syst Pharm 2009; 66(1): 82–98. 19106348 10.2146/ajhp080434

[CIT0025] Scheller D, Kolb J The internal reference technique in microdialysis: a practical approach to monitoring dialysis efficiency and to calculating tissue concentration from dialysate samples. J Neurosci Methods 1991; 40(1): 31–8. 1795551 10.1016/0165-0270(91)90114-f

[CIT0026] Schintler M V, Traunmuller F, Metzler J, Kreuzwirt G, Spendel S, Mauric O, et al. High fosfomycin concentrations in bone and peripheral soft tissue in diabetic patients presenting with bacterial foot infection. J Antimicrob Chemother 2009; 64(3): 574–8. 19578081 10.1093/jac/dkp230

[CIT0027] Scuderi G J, Greenberg S S, Banovac K, Martinez O V, Eismont F J Penetration of glycopeptide antibiotics in nucleus pulposus. Spine (Phila Pa 1976) 1993; 18(14): 2039–42. 8272956 10.1097/00007632-199310001-00019

[CIT0028] Smith J S, Shaffrey C I, Sansur C A, Berven S H, Fu K M, Broadstone P A, et al. Rates of infection after spine surgery based on 108,419 procedures: a report from the Scoliosis Research Society Morbidity and Mortality Committee. Spine (Phila Pa 1976) 2011; 36(7): 556–63. 21192288 10.1097/BRS.0b013e3181eadd41

[CIT0029] Steinkraus G, White R, Friedrich L Vancomycin MIC creep in non-vancomycin-intermediate Staphylococcus aureus (VISA), vancomycin-susceptible clinical methicillin-resistant S. aureus (MRSA) blood isolates from 2001-05. J Antimicrob Chemother 2007; 60(4): 788–94. 17623693 10.1093/jac/dkm258

[CIT0030] Stolle L B, Arpi M, Holmberg-Jorgensen P, Riegels-Nielsen P, Keller J Application of microdialysis to cancellous bone tissue for measurement of gentamicin levels. J Antimicrob Chemother 2004; 54(1): 263–5. 15190036 10.1093/jac/dkh291

[CIT0031] Tottrup M, Bibby B M, Hardlei T F, Bue M, Kerrn-Jespersen S, Fuursted K, et al. Continuous versus short-term infusion of cefuroxime: assessment of concept based on plasma, subcutaneous tissue, and bone pharmacokinetics in an animal model. Antimicrob Agents Chemother 2015; 59(1): 67–75. 25313214 10.1128/AAC.03857-14 PMC4291408

[CIT0032] Tottrup M, Bue M, Koch J, Jensen L K, Hanberg P, Aalbaek B, et al. Effects of implant-associated osteomyelitis on cefuroxime bone pharmacokinetics: assessment in a porcine model. J Bone Joint Surg Am 2016; 98(5): 363–9. 26935458 10.2106/JBJS.O.00550

[CIT0033] Whiteside L A Prophylactic peri-operative local antibiotic irrigation. Bone Joint J 2016; 98-B(1 Suppl A): 23–6. 26733636 10.1302/0301-620X.98B1.36357

